# Time-dependent reversal of synaptic plasticity induced by physiological concentrations of oligomeric Aβ42: an early index of Alzheimer’s disease

**DOI:** 10.1038/srep32553

**Published:** 2016-09-01

**Authors:** Peter Koppensteiner, Fabrizio Trinchese, Mauro Fà, Daniela Puzzo, Walter Gulisano, Shijun Yan, Arthur Poussin, Shumin Liu, Ian Orozco, Elena Dale, Andrew F. Teich, Agostino Palmeri, Ipe Ninan, Stefan Boehm, Ottavio Arancio

**Affiliations:** 1 Department of Pathology & Cell Biology, Columbia University, New York, NY, 10032, USA; 2 The Taub Institute for Research on Alzheimer’s Disease and the Aging Brain, Columbia University, New York, NY, 10032, USA; 3 Department of Neurophysiology and Neuropharmacology, Center for Physiology and Pharmacology, Medical University of Vienna, 1090, Vienna, Austria; 4 Department of Biomedical and Biotechnological Sciences, Section of Physiology, University of Catania, Catania, 95125 Italy; 5 Department of Psychiatry, NYU School of Medicine, New York 10016, USA

## Abstract

The oligomeric amyloid-β (Aβ) peptide is thought to contribute to the subtle amnesic changes in Alzheimer’s disease (AD) by causing synaptic dysfunction. Here, we examined the time course of synaptic changes in mouse hippocampal neurons following exposure to Aβ_42_ at picomolar concentrations, mimicking its physiological levels in the brain. We found opposite effects of the peptide with short exposures in the range of minutes enhancing synaptic plasticity, and longer exposures lasting several hours reducing it. The plasticity reduction was concomitant with an increase in the basal frequency of spontaneous neurotransmitter release, a higher basal number of functional presynaptic release sites, and a redistribution of synaptic proteins including the vesicle-associated proteins synapsin I, synaptophysin, and the post-synaptic glutamate receptor I. These synaptic alterations were mediated by cytoskeletal changes involving actin polymerization and p38 mitogen-activated protein kinase. These *in vitro* findings were confirmed *in vivo with* short hippocampal infusions of picomolar Aβ enhancing contextual memory and prolonged infusions impairing it. Our findings provide a model for initiation of synaptic dysfunction whereby exposure to physiologic levels of Aβ for a prolonged period of time causes microstructural changes at the synapse which result in increased transmitter release, failure of synaptic plasticity, and memory loss.

Accumulation of amyloid-β (Aβ) and tau proteins in the brain are widely accepted as key pathogenetic events in Alzheimer’s disease (AD)^1^. Specifically, elevation of soluble oligomeric forms of Aβ preceding tau deposition has been viewed as a relevant early mechanism in disease etiopathogenesis. Nevertheless, amyloid-targeted therapies have so far failed to enter the market. As a consequence, the amyloid cascade hypothesis is increasingly disputed^2^,^3^,^4^,^5^, and various other pathogenetic mechanisms are acclaimed as potential targets for novel AD treatments^2^,^3^,^4^,^6^,^7^,^8^,^9^,^10^,^11^,^12^,^13^,^14^,^15^,^16^,^17^,^18^,^19^,^20^. Despite these challenges to the role of Aβ in the disease etiopathogenesis, Aβ oligomers are clearly known to disrupt the cellular Ca^2+^ homeostasis, to cause neuronal death, to contribute to oxidative stress, and to impair synaptic function as well as plasticity^21^. Moreover, many in the field sustain that failure of the clinical trials against AD depended upon the fact that intervention occurred too late in the disease (i.e. after tau pathology has been triggered by Aβ) when it could no longer be stopped. Thus, a better understanding of the mechanisms starting Aβ toxicity in the brain might help establishing a therapy against AD.

Aβ is commonly viewed as exclusively pathological, and its physiological functions are rarely considered. The peptide has been found to be released endogenously during neuronal activity^22^,^23^ and to enhance synaptic plasticity and memory formation when administered at picomolar doses^24^,^25^,^26^ which presumably mimic the physiological concentration of the peptide in the brain^24^,^27^,^28^. Additionally, Aβ turned out to be required for normal synaptic plasticity and memory^25^,^29^,^30^. These findings lead to the question as to how the function of Aβ might switch from beneficial to harmful. Interestingly, the peptide is cleared by an enzyme, neprilysin, which declines during aging in humans as well as rodents^31^,^32^, and whose levels are reduced in the brains of AD patients^33^,^34^. Consequently, the clearance of Aβ from synaptic regions upon its release due to neural activity can be expected to abate. This, in turn, could lead to a prolonged availability of physiologic Aβ concentrations at the synapse. We therefore investigated whether the well-known facilitatory effects of picomolar Aβ on synaptic plasticity and memory^24^ might change over time. The results reveal a time-dependent reversal in the effects of picomolar Aβ on synaptic plasticity and memory, and uncover a molecular mechanism underlying the phenomenon. Together, the data support a model for the onset of AD in which synaptic dysfunction and memory loss are triggered by prolonged exposure towards picomolar concentrations of oligomeric Aβ.

## Results

### Time-dependence of the effects of picomolar Aβ on glutamate-induced plasticity in primary hippocampal cultures

**Figure 1:**
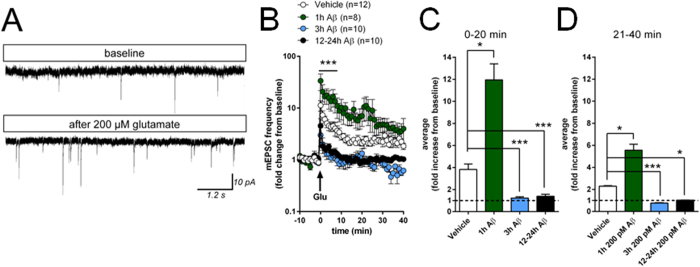
Effects of different pM Aβ exposure times on glutamate-induced synaptic potentiation. (**A**) Example traces of mEPSCs before and after glutamate (200 μM, 30 sec. in 0 Mg^2+^) perfusion. (**B**) The glutamate-induced increase in mEPSC frequency was significantly enhanced in cultures treated with Aβ_42_ for 1 hour compared with vehicle and other treatment groups. In contrast, 3 and 12–24 hour treatments with Aβ blocked glutamate-induced synaptic potentiation (main effect of Aβ treatment: *F*_(*3,9142*)_ = 111.2, P < 0.0001; main effect of time: *F*_(*50,8196*)_ = 5.9, P < 0.0001; interaction: *F*_(*150,9380*)_ = 2.3, P < 0.001, two-way ANOVA with Bonferroni post hoc test between 1 hour 200 pM Aβ and Vehicle). (**C,D**) Comparison of average increases in the first 20 min after glutamate-perfusion shows significantly increased potentiation in the 1 hour 200 pM Aβ group while potentiation was significantly reduced in the 3 hour and 12–24 hour groups (**C**). These differences remained also in the later phase of potentiation (**D**). The dotted line corresponds to the baseline values of mEPSC frequency prior to the induction of plasticity. *P < 0.05, ***P < 0.001 compared to vehicle-treated neurons; Kruskal-Wallis with Dunn’s post hoc test.

As an initial preparation to investigate the time-dependence of the effects of pM Aβ concentrations, we used primary hippocampal neuronal cultures. This preparation offers advantages over slice and *in vivo* systems, including a) visibility of synapses and cells for electrical and optical measurements; b) opportunity to identify pre- and post-synaptic neurons; c) easy access for applied drugs; d) possibility of long-term access to cells under controlled environment for biochemical manipulation. Moreover, cultured hippocampal neurons display LTP-like synaptic plasticity which can be elicited by the presence of 200 μM glutamate in Mg^2+^-free solution for 30 sec and quantified via the frequency of miniature excitatory post-synaptic currents (mEPSCs)^35^,^36^,^37^ (see an example in Fig. 1A). When we investigated the effects of 200 pM Aβ on synaptic plasticity for a variable time in 10–13 DIV cultures, we found a significant effect of Aβ treatment following exposure to 200 μM glutamate in Mg^2+^-free solution for 30 sec, with 1 hour exposure increasing plasticity, reaching significance in the first 8 min after glutamate exposure compared to vehicle (Fig. 1B). When comparing the average increases shortly after glutamate application (min 0–20, Fig. 1C), there was a significant increase in the 1 hour Aβ treated cultures. By contrast, there was a significant reduction in the 3 and 12–24 hour treated cultures compared with vehicle-treated cells (Fig. 1C). Although early synaptic potentiation eventually declines, the differences between the treatment groups conserved the same pattern at 21–40 min after glutamate-induced potentiation (Fig. 1D). Interestingly we observed a similar reduction in synaptic plasticity after 24 hr exposure to 200 nM Aβ (data not shown). Hence, the facilitation of synaptic plasticity by 200 pM Aβ is reverted into a block of long lasting synaptic potentiation when the peptide is present for extended periods of time.

### Picomolar Aβ increases the frequency of miniature neurotransmission in cultured hippocampal neurons in a time-dependent manner

**Figure 2:**
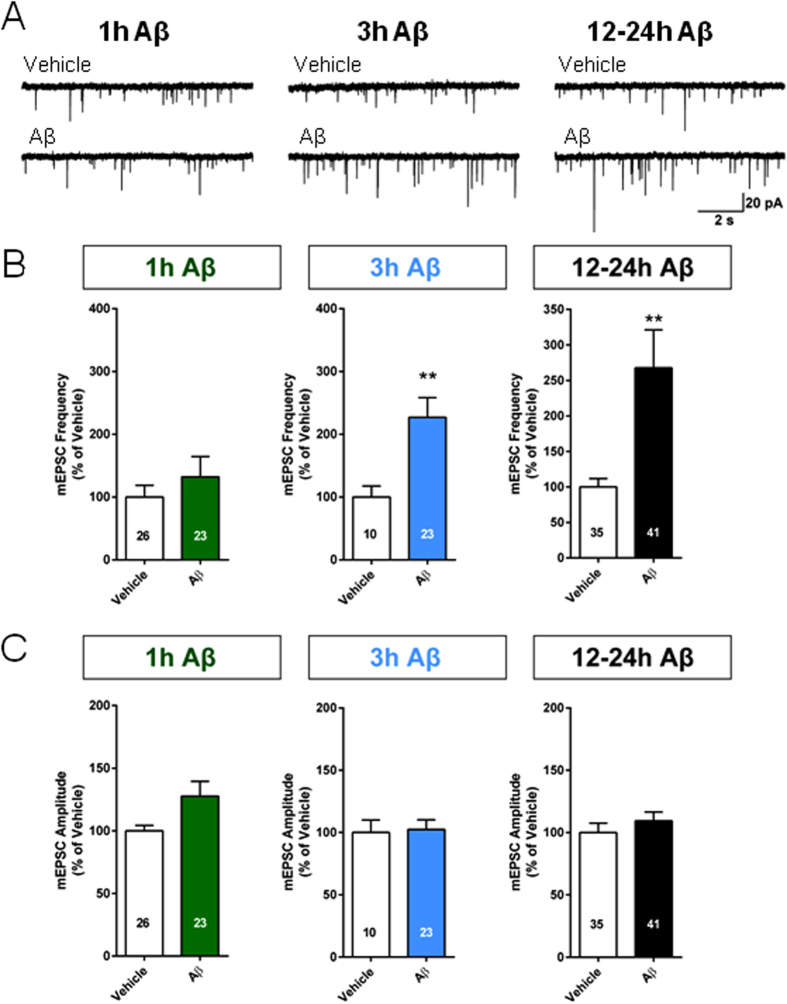
Time-dependent effects of treatment with pM Aβ on miniature neurotransmission. (**A**) Example traces of mEPSCs in vehicle- and Aβ-treated neurons after 1, 3 and 12–24 hours. (**B)** The frequency of spontaneous release of neurotransmitter was slightly increased, albeit not significantly, compared to vehicle treated cultures, after one hour (left) of perfusion with 200 pM Aβ_42_ (P = 0.581). The increase became significant after 3 (middle) (P = 0.009) and 12–24 hours (right) perfusion (P = 0.005) **P < 0.01, Mann-Whitney test. (**C**) At no time point was the amplitude of miniature events affected (P > 0.05, Mann-Whitney test).

The opposite effects of Aβ on synaptic plasticity after the different exposure periods prompted us to analyze potential changes in synaptic responses independently of the glutamate potentiation. When primary hippocampal cultures were treated with 200 pM oligomeric Aβ_42_ for variable periods of time prior to measuring mEPSCs, it became evident that their frequency was enhanced after 3 and 12 to 24 hours, but not after 1 hour (Fig. 2A,B). The increase was significant after 3 hours (Fig. 2B), and was also present at 12–24 hours after the start of Aβ-treatment (Fig. 2B). Irrespective of any period of Aβ exposure, mEPSC amplitudes remained unaltered (Fig. 2C). Such a rise in the frequency of mEPSCs can be explained either by an increase in the probability of release in existing synapses or by a conversion of silent synapses into active ones.

### Long exposures to picomolar Aβ cause an increase in the number of active boutons and faster vesicle cycling kinetics in hippocampal cultures

**Figure 3:**
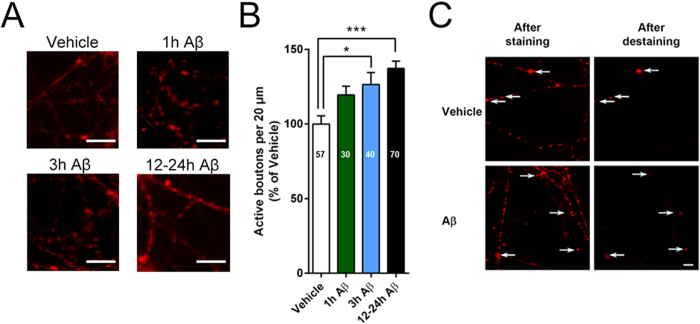
Changes in number of functional release sites and synaptic vesicle kinetics after prolonged pM Aβ exposure. (**A**) Example of FM 1–43 labeled boutons after treatment with vehicle or 200 pM Aβ_42_ for 1, 3 and 12–24 hours, scale bar = 10 μm. (**B**) The number of active presynaptic release sites was significantly increased in cultures treated with 200 pM Aβ_42_ for 3 and 12–24 hours compared with vehicle-treated cells. *P < 0.05, ***P < 0.001, one-way ANOVA with Bonferroni post hoc test. (**C**) Examples of activity-dependent FM 1–43 staining and destaining in vehicle and Aβ treated hippocampal cultures. Scale bar, 5 μm.

To examine whether the changes in mEPSC frequency were due to modifications of the release machinery at active sites or caused by the recruitment of previously silent synapses, we performed FM-dye vesicle cycling experiments. Neurons exposed to Aβ displayed a time-dependent increase in the number of active boutons which was significant after 3 and 12–24 hours (Fig. 3A,B). These results indicate that prolonged exposure to pM Aβ is associated with an increased numbers of active release sites.

The large increase in the number of “active” synapses implies two alternative possibilities, a) many boutons were not loaded in cultures that were not exposed to Aβ (i.e. these boutons were not active in normal conditions and would appear after prolonged Aβ exposure), or b) many boutons were unable to release the dye once they had taken it up in cultures that were not exposed to Aβ but release it in cultures exposed to Aβ (note that we consider active boutons those that destain after loading, thus exposure to Aβ would facilitate the release of the dye). To solve this issue, we analyzed FM staining and destaining in 3 dishes each from vehicle (number of boutons = 270) and 12–24 hour Aβ (number of boutons = 523) groups respectively (Fig. 3C). The percentage of boutons that failed to undergo destaining was 10.1% ± 2.3% and 12.2% ± 2.7%, respectively for vehicle and Aβ treated dishes of culture. Thus, Aβ-induced increase in number of active boutons does not imply enhanced destaining in Aβ-treated neurons. On the other hand, Aβ enhances appearance of new functional synapses resulting in increased neurotransmitter release.

### Prolonged exposure to picomolar Aβ is associated with an increase in the number of both pre- and post-synaptic protein clusters in primary hippocampal neuronal cultures

**Figure 4:**
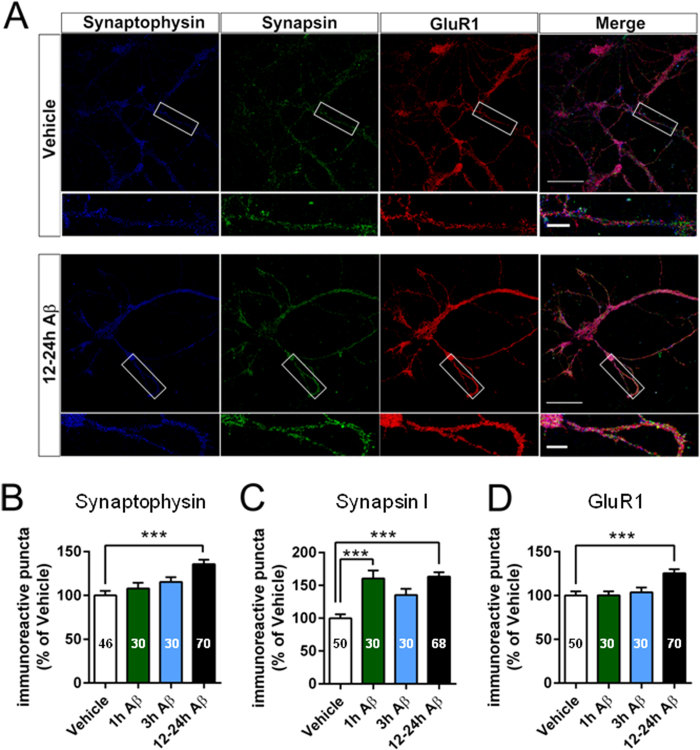
Prolonged exposure to pM Aβ changes basal distribution of synaptic proteins. (**A**) Example pictures of triple immune stainings for the pre-synaptic markers synaptophysin and synapsin I and the post-synaptic marker GluR1. White square shows region of magnification, scale bars 50 μm (upper panel) and 10 μm (lower panel). (**B**) Quantification of immunoreactive puncta for synaptophysin **C** Quantification of immunoreactive puncta for synapsin. (**D**) Quantification of immunoreactive puncta for GluR1; *P < 0.5, **P < 0.01, ***P < 0.001 compared with vehicle, Kruskal-Wallis with Dunn’s post hoc test.

Appearance of new synapses has been associated with clustering of synaptic proteins^38^. Thus, we performed immunocytochemical staining for the presynaptic proteins synapsin I and synaptophysin as well as the postsynaptic protein glutamate receptor 1 (GluR1; Fig. 4A). The number of synaptophysin-immunoreactive clusters (here referred to as puncta) per 20 μm neurite was significantly increased in 12–24 hour Aβ treated cultures compared with vehicle, whereas the 1 hour and 3 hour time points did not show significant differences compared to vehicle (Fig. 4B). Synapsin I puncta were significantly increased both at 1 hour and 12–24 hours of Aβ exposure compared with vehicle-treated cultures; an increase was also present at 3 hours (Fig. 4C), but did not reach significance, probably due to sampling size that was insufficient to reach statistical significance due to biological variation. Similarly, GluR1 puncta were significantly increased in neurons treated with Aβ for 12–24 hours compared to vehicle, whereas 1 hour and 3 hour Aβ treated cultures were not affected (Fig. 4D). These results indicate that, particularly after 12–24 hours of exposure, Aβ increases the number of pre- and post-synaptic protein clusters.

### Redistribution of synaptic proteins by prolonged exposure to picomolar Aβ is actin-dependent

**Figure 5:**
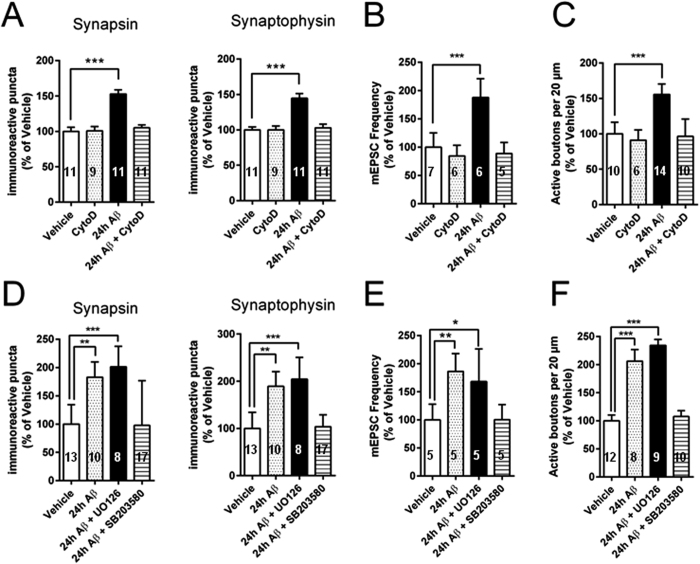
Block of actin polymerization suppresses synaptic effects of prolonged exposure to pM Aβ. (**A**) Average results from experiments in hippocampal cultures exposed to 10 nM cytochalasin D for 24 hours together with 200 pM oligomeric Aβ_42_. Block of actin polymerization re-established normal number of immunoreactive puncta for the presynaptic proteins, synapsin I and synaptophysin. (**B**) Average results from experiments in which the basal frequency of spontaneous release of neurotransmitter was measured after exposure of cultures to 10 nM cytochalasin D together with 200 pM oligomeric Aβ for 24 hours. Block of actin polymerization re-established normal neurotransmitter release. (**C**) Average results from experiments in which the number of active presynaptic boutons was measured after exposure of neurons to 10 nM cytochalasin D together with 200 pM oligomeric Aβ for 24 hours. Block of actin polymerization re-established normal number in active boutons. (**D**) Average results from experiments in which neurons were exposed to 10 μM SB203580 or 10 μM UO126 together with 200 pM oligomeric Aβ_42_ for 24 hours. Block of p38MAPK by SB203580 but not p42-p44 MAPK by UO126 re-established normal number of immunoreactive puncta for the presynaptic proteins, synapsin I and synaptophysin. (**E**) Average results from experiments in which the basal frequency of spontaneous release of neurotransmitter was measured after exposure of neurons to 10 μM SB203580 or 10 μM UO126 together with 200 pM oligomeric Aβ for 24 hours. Block of p38MAPK by 48 SB203580 but not p42-p44 MAPK by UO126 re-established normal mEPSC frequency. (**F**) Average results from experiments in which the number of presynaptic boutons was measured after exposure of neurons to 10 μM SB203580 or 10 μM UO126 together with 200 pM oligomeric Aβ_42_ for 24 hours. Block of p38MAPK by SB203580 but not p42-p44 MAPK by UO126 re-established normal neurotransmitter release. *P < 0.05, **P < 0.01, ***P < 0.001, one-way ANOVA with Bonferroni post hoc test.

The Aβ-induced appearance of new active boutons raises the question as to what might be the source of these new synapses? Synaptophysin immunoreactive puncta in cultured hippocampal neurons have been shown to undergo continual aggregation and disaggregation which both are in approximate equilibrium^38^. Given that aggregation/disaggregation of synaptic proteins is guided by cytoskeletal movements^38^,^39^,^40^,^41^,^42^,^43^,^44^, we examined whether Aβ caused cytoskeleton-mediated clustering of existing disaggregated synaptic markers. To interfere with the cytoskeleton, we used cytochalasin D, a compound that at low concentrations (10–100 nM) selectively depolymerizes actin microfilaments, a component of the cytoskeleton involved in various types of cellular motility^45^. Cytochalasin D has been found to also protect cultured rat hippocampal neurons against Aβ neurotoxicity^46^. A 24 hour treatment with 10 nM cytochalasin D had no effect on the number of immunoreactive puncta (Fig. 5A). However, the increase in synapsin/synaptophysin puncta in cultures treated for 24 hour with Aβ was absent in neurons co-treated with 10 nM cytochalasin D (Fig. 5A).

Consistent with the observation that cytochalasin D blocked the Aβ-induced increase in immunoreactivity for synapsin I and synaptophysin, this actin depolymerizing agent also prevented the increase in mEPSC frequency in cultures treated with Aβ (Fig. 5B). Cytochalasin D perfused alone did not significantly alter the mEPSC frequency (Fig. 5B), nor affected mEPSC amplitudes (cytochalasin D: 9.85 ± 1.66 pA, vehicle: 9.13 ± 1.93 pA, P > 0.05).

The numbers of functional release sites were also back to control levels following treatment of neurons with cytochalasin D in addition to Aβ (Fig. 5C). These numbers were significantly smaller than the numbers of release sites in the presence of Aβ alone and were not significantly different from those of vehicle-treated controls (Fig. 5C). In interleaved experiments, cytochalasin D perfused alone did not significantly alter the number of functional release sites (Fig. 5C). Taken together these findings show that Aβ-induced changes in synaptic protein distribution are actin-dependent. Moreover, the Aβ-induced increase in the number of functional synapses and in the frequency of mEPSCs is also actin-dependent.

p38 mitogen-activated protein kinase (p38MAPK) is a component of the pathway leading to actin polymerization^47^. Moreover it has been shown to play a role in Aβ-induced synaptic dysfunction^48^,^49^,^50^, and amounts of phospho-p38MAPK in mouse cortical cultures were found to increase about 4 folds from basal levels of 18.7 U/ng total p38 following exposure to Aβ^51^. To add further evidence in favor of the role of actin in Aβ-induced presynaptic protein redistribution, modulation of the release machinery and spontaneous release of neurotransmitter, we studied whether an inhibition of p38MAPK might affect these phenomena. Consistent with the effect of cytochalasin D, the p38MAPK inhibitor, SB203580 (10 μM), a highly selective p38MAPK inhibitor^52^, blocked the Aβ-induced increase in the number of immunoreactive puncta of synapsin I and synaptophysin (Fig. 5D). SB203580 also blocked the Aβ-induced increase in basal mEPSC frequency (Fig. 5E), as well as the rise in the number of presynaptic boutons (Fig. 5F). As a control experiment, we inhibited p42-p44 MAPK, a distinct subgroup of the MAPK family that is involved in signal transduction pathways stimulated by growth-related stimuli^53^. The effect of inhibition of p42–p44 MAPK was investigated using the MAPK kinase (MEK) inhibitor U0126 (10 μM). This concentration of U0126 is known to completely block activation of p42–p44 MAPK^54^. When we applied UO126 together with Aβ, we found that this inhibitor failed to modify the Aβ-induced increase in the number of immunoreactive puncta of synapsin I and synaptophysin (Fig. 5D), in mEPSC frequency (Fig. 5E), and in the number of presynaptic boutons (Fig. 5F). Neither SB203580 nor U0126 alone affected any of the parameters tested (data not shown). Thus, these results show that inhibition of an upstream target of actin polymerization, p38MAPK, blocks the synaptic effect of prolonged exposure to pM Aβ.

### p38MAPK inhibition re-establishes normal synaptic plasticity in cultures exposed to prolonged treatment with picomolar Aβ

**Figure 6:**
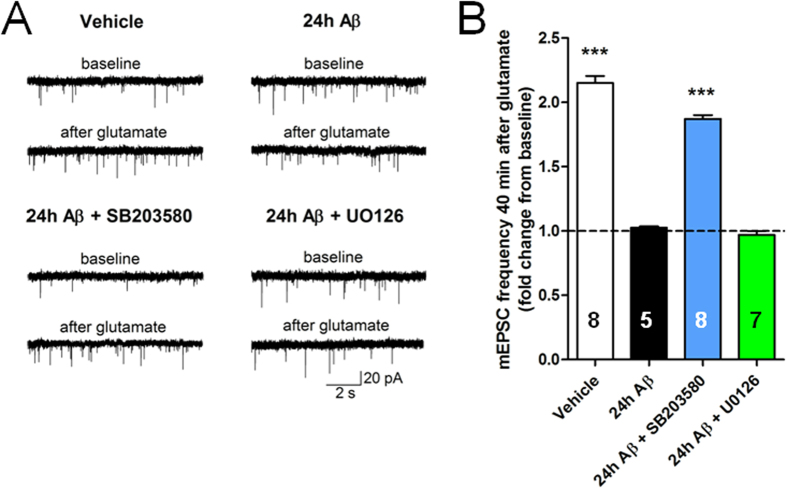
Inhibition of p38MAPK prevents impairment of synaptic plasticity by prolonged exposure to pM Aβ. (**A**) Example traces of mEPSCs at baseline and after the application of glutamate in neurons treated with vehicle, Aβ, Aβ + SB203580 or Aβ + UO126. (**B**) In cultures treated with vehicle for 24 hours, the mEPSC frequency was significantly increased 40 min after 200 μM glutamate application in Mg^2+^-free solution compared to basal conditions. This increase was not seen in cultures exposed to 200 pM Aβ for 24 hours and could be restored in neurons that where co-incubated with 10 μM SB203580 plus Aβ for 24 hours but not with 10 μM UO126 plus Aβ for 24 hours. The dotted line corresponds to the baseline values of mEPSC frequency prior to the induction of plasticity. ***P < 0.001 compared with the 24 hour Aβ group one-way ANOVA with Bonferroni post hoc test.

Rescue of synaptic protein distribution and basal neurotransmitter release by using cytochalasin D and SB203580 suggests that cells might also reacquire their ability to undergo synaptic potentiation when the pathway leading to actin polymerization is interrupted. However, there is evidence showing that proper actin polymerization is necessary for LTP as cytochalasin D is known to block LTP without affecting basal synaptic transmission^55^. Thus, to determine whether the pathway controlling actin polymerization was also involved in the effects of Aβ on synaptic plasticity, we decided to inhibit p38MAPK which, despite interfering with actin polymerization^47^, is known to ameliorate Aβ-induced synaptic dysfunction^48^,^49^,^50^. We observed that SB203580, but not U0126, reestablished glutamate induced long-lasting enhancement of mEPSC frequency in cells exposed to 200 pM Aβ for 24 hours (Fig. 6A,B). In interleaved experiments, cells that were exposed to Aβ alone failed to demonstrate synaptic plasticity compared to cells that had been exposed to vehicle. These results are consistent with the beneficial effect of p38MAPK inhibition on Aβ-induced LTP reduction in slices^48^,^49^,^50^. Taken all together, our results indicate that the detrimental effects of prolonged picomolar Aβ exposure on synaptic plasticity depend on actin polymerization.

### Time dependence of the effects of picomolar Aβ on LTP in hippocampal slices

**Figure 7:**
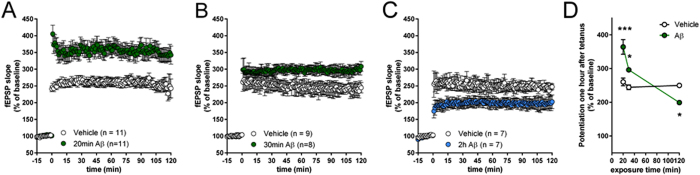
Opposite effect of pM Aβ on hippocampal LTP depending upon exposure time to the peptide. (**A,B**) 20 or 30 min exposures to 200 pM oligomeric Aβ_42_ enhanced LTP induced by theta-burst stimulation in CA3–CA1 synapses compared to vehicle treated slices. 20 min: effect of treatment: *F*_(*1,2386*)_ = 16621, P < 0.0001; effect of time: *F*_(*135,2386*)_ = 32.31, P < 0.0001; interaction: *F*_(*135,2386*)_ = 2.54, P < 0.0001; 30 min: effect of treatment: *F*_(*1,1911*)_ = 3585, P < 0.0001; effect of time: *F*_(*135,1911*)_ = 176.4, P < 0.0001; interaction: *F*_(*135,1911*)_ = 5.117, P < 0.0001; two-way ANOVA (**C**). In contrast, a prolonged 2 hour exposure to Aβ_42_ reduced potentiation. effect of treatment: *F*_(*1,1499*)_ = 3766, P < 0.0001; effect of time: *F*_(*135,1499*)_ = 78.81, P < 0.0001; interaction: *F*_(*135,1499*)_ = 4.293, P < 0.0001; two-way ANOVA (**D**). Time course of the effect of 200 pM Aβ_42_ on LTP (effect of treatment: *F*_(*1,45*)_ = 10.83, P < 0.01; effect of time: *F*_(*2,45*)_ = 23.36, P < 0.0001; interaction: *F*_(*2,45*)_ = 18.16, P < 0.0001). ***P < 0.001, *P < 0.05 two-way ANOVA with Bonferroni post hoc test.

The relationship of the cell culture results to the mechanisms of Aβ impairment in acute hippocampal slices is unknown (i.e. cultured cells might be less mature than adult ones, or cellular networks are different between cultures and adult brain). Therefore, we decided to validate findings from cell cultures on slices. As previously demonstrated^24^, short 20 or 30 min exposures to 200 pM oligomeric Aβ_42_ prior to theta-burst stimulation significantly enhanced LTP at the CA3-CA1 synapse (Fig. 7A,B). However, a prolonged treatment of 2 hours significantly reduced potentiation (Fig. 7C). In summary, a prolonged exposure to pM Aβ inverts the effect of the peptide on LTP from positive to negative (Fig. 7D). Thus, the slice preparation confirms data obtained in the cell culture system.

### Time dependence of the effects of picomolar Aβ on memory in mice

**Figure 8:**
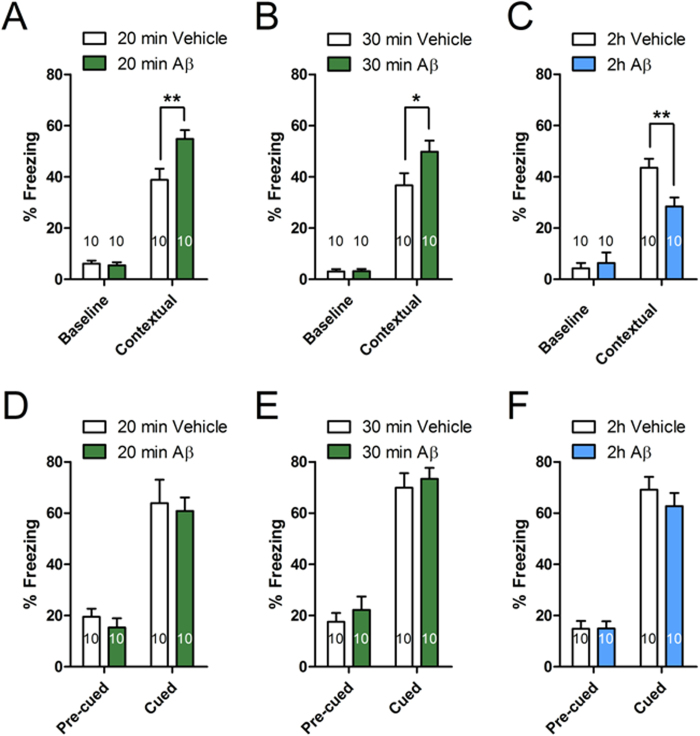
Opposite effect of pM Aβ on fear memory depending upon exposure time to the peptide. (**A,B**) Bilateral injections of 200 pM Aβ_42_ 20 or 30 min before training enhanced contextual fear memory (20 min: t = 2.88, P < 0.01; 30 min: t = 2.03, P = 0.05 compared to vehicle). (**C**) A prolonged 2-hour-exposure to 200 pM Aβ_42_ impaired contextual fear memory (t = 3.05, P < 0.01). Two-way ANOVA for time and treatment confirmed that freezing was statistically different when comparing vehicle vs. Aβ-treated mice at 20–30 min and two hours (F_(2,54)_ = 9.135, P < 0.0001) but was unaffected during the training phase among the different groups of mice (F_(2,54)_ = 0.230, P = 0.796). (**D–F**) Aβ_42_ did not modify freezing before (Pre-cued: F_(2,54)_ = 0.741, P = 0.482) or after (Post-cued: F_(2,54)_ = 0.354, P = 0.704) the auditory cue, indicating that it did not affect cued fear learning. **P < 0.01; *P = 0.05.

LTP is considered an electrophysiological surrogate of learning and memory^56^. Therefore, we decided to extend findings in cultures and slices to *in vivo* evaluation of memory. Cannulas were implanted bilaterally onto the mouse dorsal hippocampi and, after 6–8 days of recovery from surgery, animals underwent fear conditioning, a task depending upon hippocampal function. No differences were found in freezing behavior during the training phase among vehicle- or Aβ-treated mice at different time points (Fig. 8A–C). Contextual fear memory was assessed 24 hours later in response to representation of the context. As previously demonstrated^24^, a short 20 or 30 min exposure to 200 pM oligomeric Aβ prior to the electric shock enhanced contextual fear memory (Fig. 8A,B). However, a prolonged 2 hour treatment with 200 pM oligomeric Aβ induced an impairment of contextual fear memory compared to vehicle-injected mice (Fig. 8C). Twenty-four hours after contextual learning, mice were tested for cued fear learning, a type of learning depending upon amygdala function^57^. No differences were found between vehicle- and Aβ-infused mice (Fig. 8D,E), confirming that behavioral changes produced by Aβ were hippocampal dependent. Thus, as for LTP, the effect of pM Aβ on memory depends upon the exposure time, i.e. a short exposure enhancing and a prolonged exposure impairing memory.

## Discussion

This study shows that short and prolonged exposures to Aβ_42_ at concentrations that mimic endogenous levels in the healthy brain can alter synaptic properties in distinct ways. In particular, we demonstrate that while short exposures to pM concentrations of Aβ facilitate synaptic potentiation both in hippocampal cultures and slices, and enhance memory in mice, longer exposures lasting for several hours lead to reduction of synaptic plasticity and memory. These effects were associated with increased basal frequencies of spontaneous synaptic events and a higher number of functional release sites. We characterized the time course of Aβ actions on synaptic proteins and found a re-distribution of their localization over time. In addition, we found that the detrimental effects of long exposures to pM Aβ were caused by actin polymerization, as interfering with the actin machinery prevented the synaptic changes caused by prolonged exposure to pM Aβ.

Aβ treatment for 24 hours produced neurons with increased basal mEPSC frequencies, a higher number of active boutons, and increased synaptic marker density, indicating that the increased probability of spontaneous glutamate release may be due to an increased number of functional synapses. Interestingly, we also found that synaptic plasticity was impaired after 24 hours of Aβ exposure. A possible explanation could be that the newly formed synapses are unable to undergo plasticity or that previously present synapses are releasing neurotransmitter at rates that cannot be increased further. Since Aβ increased basal mEPSC frequencies, similar to glutamate-perfusion in Mg-free solution, it is also possible that release probability at previously formed synapses could not be increased any further due to similar underlying mechanisms of NMDA receptor activation and Aβ-induced modulation of neurotransmission^58^.

Cultured hippocampal neurons treated with 200 pM Aβ for one hour showed increased glutamate-induced plasticity while, surprisingly, basal miniature neurotransmission frequency and vesicle release properties were not affected. These results are in agreement with the observation in slices that a brief exposure to pM Aβ increases synaptic plasticity without affecting basal probability of neurotransmitter release^24^. Consistent with these observations, CREB-regulating micro RNAs can alter synaptic plasticity without altering the probability of neurotransmitter release in hippocampal neurons^59^. Thus, it is likely that Aβ enhances transmitter release only during the induction of plasticity without affecting basal release. In support of this conclusion, post-tetanic potentiation, a type of synaptic plasticity corresponding to a transient increase of glutamate release from the presynaptic terminal during its induction^60^, was found to be increased in slices after short exposure to pM Aβ^24^.

Reversal of Aβ-induced alteration in the synaptic machinery by inhibition of p38MAPK, an upstream target for actin polymerization, but not by inhibition of p42-p44 MAPK, a distinct subgroup of the MAPK family, supports the notion that Aβ-induced effects on synaptic function are mediated by actin polymerization. Consistent with this observation, there are multiple lines of evidence indicating that actin is involved in AD: (i) the presence of polymerized actin aggregates, also known as actin stress fibers, in the hippocampi of AD patients^61^; (ii) the appearance of actin stress fibers following Aβ exposure in cultured hippocampal neurons due to the activation of the p38MAPK/MAPK-associated-protein-kinase-2/heat-shock-protein-27 signaling pathway^62^; (iii) the prevention of the Aβ-induced LTP block by inhibition of p38MAPK^48^,^49^,^50^; (iv) the protection of cultured hippocampal neurons against Aβ neurotoxicity by inhibition of actin polymerization^46^,^63^,^64^. Taken together these results indicate that, following Aβ exposure, pre-existing synaptic proteins which are genuinely expressed within the cell, but not aggregated to form puncta, would move to converge on new spots and form new puncta underlying the formation of functional synapses.

We have observed that, in addition to the presynaptic proteins synaptophysin and synapsin I, the postsynaptic protein GluR1 undergoes changes in distribution following prolonged exposure to pM Aβ. This finding suggests that the peptide causes coordinated changes in distribution of a wide array of synaptic proteins. Such a phenomenon would be necessary for the formation of fully functional synapses. Consistent with this interpretation, a series of concomitant microstructural changes due to protein redistribution and clustering at synaptic domains have been described to occur during plasticity^65^. Thus, Aβ would hijack mechanisms of normal synaptic plasticity to initiate synaptic dysfunction, and impair memory.

Can prolonged exposure to pM concentrations of Aβ occur at the synapse? One can envision situations such as metabolic stress that lead to changes in the processes that regulate the homeostasis of Aβ at the synapse^66^. Alternatively, Aβ downstream effectors might develop resistance to the peptide, resulting in a demand for more Aβ. Accordingly, overproduction of Aβ or loss of its removal may become constitutive and result in synaptic dysfunction. The Aβ clearance enzyme neprilysin might also play a key role. Neural activity has been found to contribute to Aβ clearance in a neprilysin-dependent manner^67^. Normal aging leads to decreased neprilysin levels in the hippocampus both in humans and rodents^31^,^32^; (and our own observation in mice). Furthermore, AD brains show decreased neprilysin compared to age-matched control brain^33^,^34^. This raises the possibility that Aβ may be less effectively cleared in the brains of elderly individuals during neural activity, and that this deficit becomes exacerbated in AD.

The task of elucidating the mechanisms that eventually lead to synaptic dysfunction is in part complicated by difficulties in providing direct access of drugs to the synapse using conventional systems such as the hippocampal slice or *in vivo* preparation. Primary culture systems provide a powerful tool to overcome this problem and to study synaptic function. However, differences might exist between cultures and more conventional systems (i.e. cultured cells might be less mature than adult ones, or cellular networks are different between cultures and adult brain). These differences together with difficulties in having direct access to the synapse might explain subtle discrepancies regarding the extent and the time course of the reduction of potentiation after prolonged Aβ exposure in cultures vs. slices (i.e. block of plasticity in cultures treated with Aβ was complete at 3 hours and 24 hours, whereas slices perfused with Aβ for 2 hours had a partial block of plasticity). Nevertheless, the overall finding that synaptic potentiation is analogously altered in both, cultures and slices by pM concentrations of Aβ justifies the use of cell cultures to gain insight on the synaptic dysfunctions present in AD.

It is well known that augmented release of glutamate can cause neurotoxicity and eventually neuronal cell death^68^. Here, we propose a model of Aβ-induced synaptic dysfunction in which the extended presence of Aβ_42_ will lead to a bell-shaped time course of synaptic marker redistribution and synaptic dysfunction: the initial increase in glutamate transmission as shown here will result in excitotoxicity which will finally cause neuronal degeneration and loss of synaptic markers as reported in several previous studies^69^. Consistent with this model, long-term inhibition of Aβ degradation through treatment of cultured hippocampal neurons with the neprylisin inhibitor thiorphan for 48 hours leads to loss of synapses^58^. Intriguingly, it has been shown that neuronal activity boosts Aβ_42_ production^22^. Thus, the Aβ-induced increase in glutamate release can be expected to raise neuronal activity, this, in turn, will further Aβ_42_ production which again supports glutamate release thereby establishing fatal *circulus vitiosus*. Accordingly, restricting the release of glutamate during early phases of the disease can be envisioned to delay the course of the pathology.

Glutamate excitotoxicity is a well-known key event in the pathophysiology of numerous neurological and psychiatric diseases and culminates in synapse loss and nerve cell death^68^. The above results point towards excitotoxicity due to Aβ acting via a p38MAPK-dependent signaling cascade as early pathogenetic mechanism in the development of AD. In support of this conclusion, *in vivo* studies using selective p38αMAPK inhibitors reported improvement of neuroinflammation, synaptic dysfunction, and memory loss in animal models of AD^48^,^49^,^50^. Together, these previous and the present results pinpoint this kinase as a promising therapeutic target in AD.

## Methods

### Animals

All protocols involving animals were approved by Columbia University and the Institutional Animal care and Use Committee (IACUC); experiments involving animals were performed in accordance with the relevant approved guidelines and regulations. C57BL/6J mice were taken from a colony kept at the animal facilities of Columbia University and University of Catania. They were maintained on a 12 hour light/dark cycle in temperature- and humidity-controlled rooms. Food and water were available *ad libitum*.

### Primary hippocampal cultures

Primary hippocampal cultures were prepared at postnatal day (PD) 0-1 as described previously^70^. Briefly, animals were decapitated, the brain was rapidly excised and placed into ice-cold Hanks Buffered Saline Solution (HBSS, Thermo Fisher Scientific, MA, USA). Hippocampi were removed and digested for 15 min at 37 °C in minimal essential medium (MEM, Thermo Fisher Scientific) containing 0.25% of trypsin. Thereafter, tissue was transferred to DMEM+GlutamMAX (Thermo Fisher Scientific) containing 10% fetal calf serum (FCS) and gently disrupted using a flame-polished Pasteur pipette. Following centrifugation at 4 °C for 8 min at 800 RPM, cells were resuspended in fresh DMEM+GlutaMAX +10% FCS and plated on coverslips coated with poly-D-lysine and laminin at a density of approx. 50 000 cells/coverslip. After 12–24 hours, medium was aspirated and replaced with pre-warmed Neurobasal A medium containing B27 containing 1% FCS, L-glutamine (400 μM), kynurenic acid (KA, 500 μM), 5-Fluoro-2′-deoxyuridine (FDU, 25 μM) and β-Mercaptoethanol (24.5 μM).

### Oligomeric Aβ preparation and culture treatment

Aβ oligomers were prepared as described previously^71^. Initially, a protein film was prepared by dissolving Aβ_42_ lyophilized powder (American Peptide, CA, USA) in 1,1,1,3,3,3-Hexafluoro-2-Propanol (HFIP) and subsequent incubation for 2 hours at room temperature to allow complete monomerization. Then, a clear peptide film was obtained by vacuum centrifugation in a SpeedVac centrifuge at 800 g which was stored at −80 °C and used within 6 month. The Aβ film was dissolved in DMSO under sonication for 10 min to obtain a concentration of 5 mM. To start oligomerization, 98.4 μl of sterile artificial cerebrospinal fluid (ACSF, concentrations in mM: NaCl 125, NaHCO_3_ 25, KCl 2.5, Glucose 25, NaH_2_PO_4_ 1.25, MgCl_2_ 1, CaCl_2_ 2) was added to 1.6 μl of 5 mM Aβ solution and incubated for 12 hours at 4 °C. This sterile, oligomerized Aβ solution was then diluted to a final concentration of 80 nM in ddH_2_O. For each coverslip, 2.5 μl of 80 nM Aβ solution was added to 200 μl of culture medium taken from each well, thoroughly mixed, and pipetted drop-by-drop back into the well for a final concentration of 200 pM Aβ. Vehicle treated cultures only received DMSO/ddH_2_O for the same duration as Aβ treated cells. In the investigation of effects of Aβ on basal neurotransmission, vehicle treatment was always performed in the same batch of culture dishes to allow for adequate comparison of Aβ exposure effects.

### Electrophysiology on cell cultures

Pyramidal-shaped hippocampal neurons at 10–13 days *in vitro* (DIV) were recorded using the whole-cell patch clamp technique, as previously described^35^,^72^. One coverslip was transferred to the recording chamber (RC-26GLP, Warner Instruments, CT, USA) and cells were visualized with a TS100 ECLIPSE microscope (Nikon, Tokyo, Japan). A PC-501A amplifier (Warner Instruments) and a 1322A Digidata (Molecular Devices, CA, USA) were used to acquire electrophysiological recordings with pClamp 10 software (Molecular Devices). Signals were filtered at 1 kHz and sampled at 10 kHz. Glass pipettes with a resistance of 4–6 MΩ were generated using a PIP5 horizontal pipette puller (HEKA, NY, USA). The intracellular solution contained (in mM): K-gluconate 130, KCl 10, EGTA 0.6, HEPES 5, MgCl_2_ 5, CaCl_2_ 0.06, MgATP 4, Na_2_GTP 0.3, Phosphocreatine 20, Leupeptine 0.2, pH = 7.1 (adjusted with KOH) and 310 mOsm/kg (adjusted with sucrose). The bath solution consisted of (in mM): NaCl 119, KCl 5, HEPES 20, Glucose 30, MgCl_2_ 2, CaCl_2_ 2, pH 7.4 (adjusted with NaOH) and 310 mOsm (adjusted with sucrose). To record miniature excitatory postsynaptic currents (mEPSCs), tetrodotoxin (TTX, 1 μM, Ascent Scientific, Cambridge, MA, USA) and picrotoxin (PTX, 100 μM) were added to the bath solution. In order to investigate synaptic plasticity, Mg^2+^-free bath solution containing glycine (1 μM) was perfused for 1 min followed by the application of the same solution containing glutamate (200 μM) for 30 sec. Subsequently, perfusion was resumed with standard Mg^2+^-containing bath solution. In experiments using Aβ treated cultures, Aβ oligomers (200 pM) were also added to the bath solution in order not to interrupt treatment duration and this time was taken into account for the calculation of the exposure period.

### Vesicle Cycling

Following the transfer of a coverslip containing primary hippocampal neurons at 10–13 DIV into the recording chamber, the chamber was mounted on an Olympus IX81 microscope (Olympus, PA, USA) perfused with normal bath solution containing (in mM): NaCl 119, KCl 2.5, HEPES 25, glucose 30, sucrose 28.5, CaCl_2_ 2, MgCl_2_ 2. In addition, 10 μM CNQX and 50 μM D-AP5 were added to the bath solution to inhibit the activation of AMPARs and NMDARs during electrical stimulation. Vesicle loading was performed as previously described^35^. Briefly, FM 1–43 was loaded by changing the perfusion medium from normal saline bath solution to hyperkalemic bath solution (31.5 mM NaCl, 90 mM KCl, 2 mM CaCl_2_, 2 mM MgCl_2_, 25 mM HEPES and 30 mM glucose) with 5 μM FM 1–43 for 45 seconds. The perfusion solution was then changed back to normal bath solution for 10 minutes to wash off the dye from the external medium. ADVASEP-7 (1 mM, CyDex, Inc., Overland Park, KS), an anionic cyclodextrin complexing agent was introduced for 60 seconds in the washing bath solution at 1 and 6 minutes of washing for enhanced removal of the dye from the external medium. After 10 min wash period, which was sufficient for the complete recycling and repriming of the dye-stained population of synaptic vesicles, an image was taken to record the loading of FM 1–43 in the synaptic boutons. The culture was then exposed to multiple 15 seconds application of hyperkalemic bath solution (without FM 1–43) to evoke repeated cycles of exocytosis, which facilitated release of the dye from the vesicles. An image was taken after 30 minutes of repeated cycles of exocytosis and washing with normal bath solution. The difference between the images before and after multiple exposures to hyperkalemic solution gave the measure of FM 1–43 stained vesicles.

### Immunocytochemistry

Cells (10–13 DIV) were fixed with 4% PFA in 20% sucrose, as previously described^65^. Staining was performed using mouse anti-synapsin1 (1:300; BD Bioscience, CA, USA), rabbit anti-GluR1 (1:300; Merck, KGaA, Germany) and Oyster-650 conjugated mouse anti-synaptophysin (1:200; Synaptic Systems GmbH, Germany). Secondary antibodies were Dylight594-labeled goat anti-rabbit and Dylight488-labeled goat anti-mouse (1:800, Jackson ImmunoResearch Laboratories, Inc., PA, USA). Pictures were captured using a Nikon A1R MP confocal microscope with a 60x/1.49 NA Apo-TIRF objective.

### Slice preparation and recordings

Hippocampal slices were cut as previously described^73^. Briefly, 3–4 month old animals were sacrificed and their hippocampi extracted. 400 μm slices were obtained and maintained in an interface chamber continuously perfused with a solution (ACSF) consisting of (in mM): 124.0 NaCl, 4.4 KCl, 1.0 Na_2_HPO_4_, 25.0 NaHCO_3_, 2.0 CaCl_2_, 2.0 MgCl_2_, and 10.0 glucose, and bubbled with 95% O_2_ and 5% CO_2_. Extracellular recordings were performed by stimulating the Schaeffer collateral fibers and recording in CA1 stratum radiatum with a glass electrode filled with ACSF. Following recording of a 15 min baseline, LTP was evoked through a theta-burst stimulation (four pulses at 100 Hz, with the bursts repeated at 5 Hz and each tetanus including three 10-burst trains separated by 15 sec). Slices were perfused with Aβ_42_ administered through the bath solution until the application of the theta-burst.

### Infusion technique

After anesthesia with Tiletamine + Zolazepam (60 mg/kg) and Medetomidine (40 μg/kg), mice were implanted with a 26-gauge guide cannula into the dorsal part of the hippocampi (coordinates: posterior = 2.46 mm, lateral = 1.50 mm to a depth of 1.30 mm). After 6–8 days of recovery, 200 pM Aβ or vehicle were infused bilaterally in a final volume of 1 μl over 1 min through infusion cannulas that were connected to a microsyringe by a polyethylene tube. Mice were injected 20 or 30 min before the training session of the fear conditioning test. For the prolonged exposure, mice were infused every 30 min for four times. During infusion, animals were handled gently to minimize stress. After infusion, the needle was left in place for another minute to allow diffusion. In some animals, after behavioral studies, a solution of 4% methylene blue was infused for localization of infusion cannulas.

### Fear conditioning

Fear conditioning was performed as previously described^24^. During the first day, mice were placed in the conditioning chamber for 2 min before the onset of a discrete tone [conditioned stimulus (CS)] (a sound that lasted 30 s at 2800 Hz and 85 dB). In the last 2 sec of the CS, mice were given a foot shock [unconditioned stimulus (US)] of 0.70 mA for 2 sec through the bars of the floor. After the CS/US pairing, the mice were left in the conditioning chamber for 30 sec and then they were placed back in their home cages. Freezing behavior, defined as the absence of all movement except for that necessitated by breathing, was manually scored. During the second day, we evaluated the contextual fear learning. Mice were placed in the conditioning chamber and freezing was measured for 5 consecutive min. During the third day, we evaluated the cued fear learning. Mice were placed in a novel context (rectangular black cage with vanilla odorant) for 2 min (pre-CS test), after which they were exposed to the CS for 3 min (CS test), and freezing was measured. Sensory perception of the shock was determined through threshold assessment.

### Drugs and chemicals

Unless stated otherwise, drugs and compounds were purchased from Sigma Aldrich (MO, USA).

### Data analyses and statistics

For electrophysiological recordings on cultures, mEPSC traces were analyzed using MiniAnalysis software (Synaptosoft Inc., NJ, USA) and subsequently manually controlled to assure correct detection of events. mEPSC data is presented as % of the vehicle that was recorded on the same day from the same culture batch, to eliminate variations produced by inadvertent differences between culture batches. Vesicle cycling data was analyzed using ImageJ software. For the vesicle cycling experiments, the percentage of FM 1–43 fluorescence was calculated based on the fluorescence intensity of presynaptic boutons immediately before the application of the destaining stimulus. The number of active boutons was determined in ImageJ along a measured length of 20 μm at 12 μm from the cell body in several randomly selected processes per dish. The boutons which showed a reduction in fluorescence after electrical stimulation were considered active. Similarly, immunocytochemically labeled puncta were analyzed along 20 μm of randomly selected processes (~10 processes/coverslip) in ImageJ. For electrophysiological recordings on slices, results were analyzed in pClamp 10 (Molecular Devices). For behavioral studies, freezing was manually scored and results were analyzed in Systat 9 (Chicago, IL, USA). For these experiments on adult mice sexes were balanced across groups. Statistical analyses and graph preparation were performed in Prism 5 (GraphPad Software, Inc., La Jolla, CA, USA). For normally distributed data parametric tests were performed; in case of non-Gaussian distribution non parametric tests were performed. In some experiments, each single data point was normalized to the mean of the vehicle, allowing for the expression of SEM also of the vehicle group. In order to avoid errors due to operator judgment, all images were subjected to exactly the same acquiring and analysis conditions. All data is presented as mean ± SEM and statistical significance was considered when p < 0.05.

## Additional Information

**How to cite this article**: Koppensteiner, P. *et al*. Time-dependent reversal of synaptic plasticity induced by physiological concentrations of oligomeric Aβ42: an early index of Alzheimer’s disease. *Sci. Rep.* **6**, 32553; doi: 10.1038/srep32553 (2016).
